# Bibliometric Analysis of the Evolution and Distribution of Research on Analytical Methods for Climate-Sensitive Infectious Diseases in Latin America and the Caribbean

**DOI:** 10.3390/ijerph22121834

**Published:** 2025-12-08

**Authors:** Sebastian Castano-Duque, Sergio Cuellar, Catalina González-Uribe, Camila González, Juliana Helo, Natalia Nino-Machado, Monica Pinilla-Roncancio

**Affiliations:** 1 Hospital Universitario San Ignacio, Cra. 7 N° 40–62, Bogotá 110231, Colombia; sebastian_castano@javeriana.edu.co; 2 Knowten S.A.S, Calle 65 B 88 59, Bogotá 110231, Colombia; sergisan2011@gmail.com; 3 Sustainable Development Goals Research Centre, Universidad de Los Andes, Cra. 1 N° 18a-12, Bogotá 111711, Colombia; cgonzalez@uniandes.edu.co (C.G.-U.); n.nino58@uniandes.edu.co (N.N.-M.); 4 Centro de Investigaciones en Microbiología y Parasitología Tropical, CIMPAT, Departamento de Ciencias Biológicas, Universidad de los Andes, Cra. 1 N° 18a-12, Bogotá 111711, Colombia; c.gonzalez2592@uniandes.edu.co; 5 School of Economics, Universidad de los Andes, Cra. 1 N° 18a-12, Bogotá 111711, Colombia; j.helo@uniandes.edu.co; 6 School of Medicine, Universidad de los Andes, Cra. 1 N° 18a-12, Bogotá 111711, Colombia

**Keywords:** climate-sensitive infectious diseases, bibliometric analysis, Latin America and the Caribbean

## Abstract

Climate-Sensitive Infectious Diseases (CSIDs) are diseases whose prevalence and transmission are heavily influenced by climatic factors, posing a significant challenge to public health, particularly in vulnerable regions such as Latin America and the Caribbean (LAC). This study employs a bibliometric analysis to evaluate the evolution and distribution of research on CSID and the analytical methods employed in the field. Using bibliometric and text-mining techniques, the analysis examines publication trends, research hotspots, and methodological developments from 2015 to 2024. The results highlight a regional concentration of research, with Brazil leading in CSID studies, particularly on arboviruses such as dengue, Zika, and chikungunya. The analysis also reveals the predominance of regression models, time-series analysis, and spatial analysis as primary methods used to forecast and analyze disease outbreaks. However, advanced techniques such as neural networks and niche modeling are gaining traction, indicating a shift towards more data-intensive approaches. The findings underscore the importance of enhancing forecasting capabilities and integrating analytical models into public-health systems to anticipate the impact of climate change on disease patterns. This study offers critical insights into methodological trends and identifies gaps for future research, contributing to more effective decision making in public health across Latin America and the Caribbean.

## 1. Introduction

Many infectious diseases are sensitive to changes in climatic variables such as temperature, precipitation, and humidity, which affect their prevalence, incidence, and distribution [1]. As a result, climate change is worsening the global burden of infectious diseases and poses a significant threat to health security [2], particularly in developing regions. Latin America and the Caribbean (LAC) is especially vulnerable due to its dependence on natural resources, geographic characteristics, and persistent socioeconomic inequalities [3].

The term Climate-Sensitive Infectious Disease (CSID) refers to infections whose transmission and spread are directly influenced by climatic and environmental conditions, including water-borne, food-borne, soil- and dust-associated, zoonotic, and vector-borne diseases. The largest epidemics reported in the LAC region in the past decade were caused by mosquito-borne diseases. These diseases include dengue, Zika, chikungunya, yellow fever, leishmaniasis, malaria, Chagas disease, Rickettsiosis, and schistosomiasis [4]. Nearly 12 million dengue cases were recorded in the Americas in 2024, showing an increase of 204 per cent compared with the same period in 2023 (4.6 million), and 381 per cent compared with the average of the last five years [5]. Temperature can affect the temporal and spatial distribution of mosquitoes, as well as pathogen development and survival [6]. In the case of malaria, *Anopheles* mosquitoes have changed their behavior and increased their survival rate due to higher temperatures, humidity, and rainfall. Extreme weather events such as heat waves and floods can directly affect transmission and disease burden.

Understanding advances in the analysis of CSID is crucial for identifying the infections most affected by climate change, developing targeted protective measures and preparedness plans for vulnerable regions, and responding to international calls, such as those from the World Health Organization (WHO), to address the health impacts of climate change [7]. Recent advances in forecasting extreme climatic events have spurred growing interest in developing predictive models to anticipate CSID risks. However, the evidence linking extreme climate events to CSID outbreaks has yet to be fully collated and synthesized [8]. Therefore, scientific research that enhances forecasting capabilities and enables us to anticipate the effects of environmental conditions on disease patterns is highly sought after for the purposes of public-health planning and early warning [8]. These systems typically employ statistical methods and predictive models to estimate infectious-disease outbreaks, which may involve forecasting various aspects of disease activity, such as the timing, severity, or geographic location of outbreaks, depending on specific objectives [6]. Consequently, analyzing the most frequently used analytical methods in the field of CSID is essential for understanding the foundation of future capabilities to integrate these models into structured information systems for informed decision making.

CSID as a topic has become an important issue, of worldwide interest [9], as reflected in the rising trends of research publications studying the phenomenon [7]. Some reviews, scoping reviews, and bibliometric analyses have been published with the aim of understanding the dynamics of scientific publications. From a broad perspective, a bibliometric analysis assessed research activity in climate change and infectious diseases, providing researchers and policymakers with baseline data in this field by analyzing publications from 1980 to 2019 [7]. Another bibliometric analysis on the prediction of infectious diseases was conducted to assess the status and research hotspots objectively, using global data. The results revealed a growing trend in the number of documents, indicating an increasing interest and attention to this field by experts and scholars each year [10].

In the LAC region, a study reviewed scientific articles linking vector-borne diseases to climate variability, using spatial analysis to identify geographic regions where data were lacking [11]. This approach, though uncommon in reviews, effectively compared disease distribution with climate maps and highlighted local clusters. However, none of the mentioned analyses focuses primarily on the publications concerning the analytical methods used to study CSID in LAC countries. Therefore, the main purpose of this research is to evaluate the evolution and distribution of research on CSID and associated analytical methods in the context of the LAC region. For this purpose, we use bibliometric methods that enable us to document empirically the volume, the intellectual structure, and the knowledge-development directions in the field. Using a combination of bibliometric, text-mining, and visualization analyses, we address the following research questions. How has the research on information systems that include Climate-Sensitive Infectious Diseases information progressed in LAC countries over the past ten years? How has the volume of scientific publications on different CSIDs varied between LAC countries? And what is the distribution of research across different CSIDs and the analytical methods used to study them?

## 2. Methods

The methodology employed in this study integrates bibliometric analysis with advanced text-processing techniques through a systematic and transparent workflow aligned with science-mapping principles. Bibliometrics provides quantitative tools to examine scientific output, while science mapping allows for the exploration of the conceptual structure and evolution of research fields over time [12].

By combining bibliometrics with Natural Language Processing (NLP) techniques, it is possible to uncover hidden thematic patterns and analytical tendencies not visible through traditional approaches [13]. Hybrid clustering methods that combine bibliographic coupling—an approach that groups documents based on shared references—with text mining can more effectively identify and label emerging research topics [14,15]. The use of word-embedding representations further enhances topic extraction and often outperforms more conventional text-analysis techniques [16].

**Figure 1:**
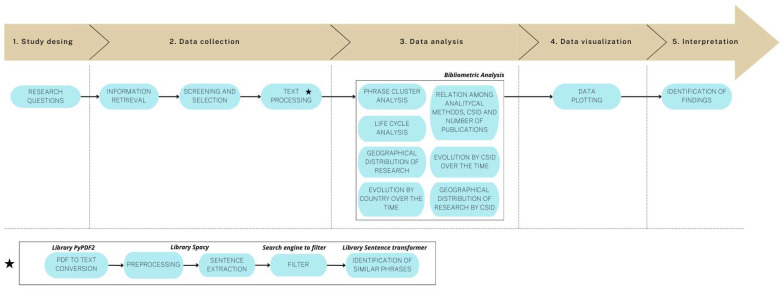
Methodology phases of the study. The start represents that the graph continues there.

We followed five phases, which are presented in Figure 1: (1) Study design, (2) Data collection, (3) Data analysis, (4) Data visualization, and (5) Interpretation [17].

### 2.1. Study Design

We formulated the specific research questions and designed a search strategy using search terms that combined CSID with analytical methods and climate-related terms, including the names of LAC countries. As each database and platform has a specific syntax, the search strategies were adjusted to fulfil the requirements of each database. Appendix A, Appendix B, Appendix C, Appendix D, Appendix E, Appendix F, Appendix G, Appendix H, Appendix I, Appendix J and Appendix K presents the search strategies used for each database. The search-time window was from 2015 to February 2024. We chose 2015 because of the significance of the Paris Agreement [18].

### 2.2. Data Collection

Searches were conducted in scientific databases (Scopus, Web of Science, PubMed), regional databases (Virtual Health Library—VHL, LILACS) and gray-literature sources, including repositories of the Inter-American Development Bank (IDB), Development Bank of Latin America (CAF), and the Economic Commission for Latin America and the Caribbean (ECLAC). Additional searches included arXiv, ProQuest Dissertations and Theses, MedRxiv, and BioRxiv.

Although this is not a systematic or scoping review, we used organizational elements from [19] to structure the workflow for a relevance assessment of the documents. Likewise, we used a PRISMA diagram to transparently document the flow of retrieved records, and the process was adapted to the needs of a bibliometric and text-mining study rather than a systematic review [20]. Filtering was based on relevance to the study’s analytical focus, not on methodological quality. Two authors independently conducted the filtering workflow using the Covidence platform.

Because the purpose of this study was to analyze how analytical models are used to assess, characterize, or predict CSID, documents were included only if they applied an analytical, statistical, or computational model to human infectious-disease outcomes influenced by climate variability in at least one LAC country. Therefore, studies that focused solely on vectors, climate–vector interactions or environmental conditions without epidemiological data on human cases were excluded, as they could not inform our objective. Descriptive reports and conceptual papers were also removed because they do not employ modeling frameworks relevant to understanding CSID behavior. These criteria ensured that the final dataset contained only studies with empirical human-health outcomes, allowing us to identify methodological patterns in CSID research with consistency and precision.

Full texts were imported in PDF format and converted to machine-readable text using the PyPDF2 Python library 4.0. This conversion was essential for transforming static documents into malleable text suitable for computational analysis. We included bibliographic information obtained from Covidence in CSV format to complement the data. The text was then pre-processed to ensure consistency. Pre-processing steps included tokenization, lower-casing, stop-word removal, eliminating common stop words, and stemming or lemmatization, which reduces words to their base or root form [21]. Following pre-processing, we carried out sentence extraction, segmenting each document into individual sentences while preserving semantic coherence. The SpaCy Python library 3.7 was used for both pre-processing and sentence segmentation because of its capacity to recognize linguistic structure and accurately identify sentence boundaries [22]. To ensure the relevance of the sentences to the main topics of CSID and analytical techniques, we applied a keyword-based filtering step. Using the controlled vocabulary listed in Appendix J, a search engine matched sentences against predefined terms. Only sentences containing at least one key term were retained for further analysis. All unrelated sentences were removed. This procedure ensured that the final dataset reflected the thematic scope of the study and excluded extraneous material not pertinent to the analytical objectives.

As a preliminary step to clustering, we identified similar phrases using sentence embeddings combined with t-Distributed Stochastic Neighbor Embedding (t-SNE) for dimensionality reduction [22,23]. Sentence embeddings were generated using the Sentence Transformers Python library 4.37, which represents each sentence as a vector in a continuous space that captures its contextual meaning. After generating these vectors, we applied t-SNE to reduce their dimensionality for visualization in two dimensions (t-SNE dimension 0 and t-SNE dimension 1).

To identify information related to CSID, analytical methods, and country-specific content within each publication, we applied Named Entity Recognition (NER) [24]. Using this tool, we classified the text elements in the full text and employed a text-matching method to identify the publications that were mainly related to the three categories (CSID, analytical methods, and countries). For our classification process, we established a rule of thumb: a keyword must appear at least three times within the text to classify the publication under the relevant category. This frequency threshold was chosen to ensure the significance and relevance of the classification. This threshold helped minimize misclassification due to incidental mentions. The vocabulary in Appendix J guided this process, and we conducted a manual validation step to confirm that classifications were contextually appropriate. Because publications often discuss more than one disease, the total keyword count may exceed the number of articles.

All text-processing steps of the method were performed using the advanced analytical tool KNIME 5.2^®^. The Konstanz Integration Miner, or KNIME^®^, is an open-source data-pipelining and analytics platform that enables the creation of automated workflows for processing, transforming, analyzing, and visualizing complex datasets [25]. Its use in related bibliometric studies has been documented across various research domains [26,27,28,29].

### 2.3. Data Analysis and Visualization

#### 2.3.1. Phrase-Cluster Analysis

To examine the relationships between analytical methods and CSID, we conducted a phrase-cluster analysis. Clustering similar phrases reveals connections and thematic regularities in the document analysis [30], which enables us to understand the main concepts of a group of research articles. The two-step framework, incorporating a word-embedding scheme and cluster analysis, improves topic extraction from the scientific literature, outperforming conventional methods [16]. The resulting clusters were visualized in a two-dimensional plot, with t-SNE dimension 0 on the x-axis and t-SNE dimension 1 on the y-axis. Because the t-SNE analysis was performed at the sentence level and the workflow did not retain identifiers linking each sentence to its source article, it is not possible to determine how many individual publications contributed to each thematic cluster.

#### 2.3.2. Life-Cycle Analysis

To understand changes in the volume of CSID-related publications in LAC over time, we developed a life-cycle analysis using annual research output. The life-cycle (or S-shaped curve) approach helps identify common developmental stages in a scientific field. Our analysis followed the framework proposed by Ernst [31] and classified each field’s trajectory into creation, adoption, peak, or decay phases, according to the conceptual model of Singh et al. (2022) [32].

#### 2.3.3. Geographical Distribution of Research

To assess how CSID research has progressed across LAC countries over the past decade, we examined the geographic distribution of publications using the classifications generated. This bibliometric assessment shows the spread and concentration of research activity related to CSID and analytical techniques. By analyzing the countries of origin of the publications, we identified regions with higher research intensity and leading contributions. Results were visualized using a map of LAC, with point sizes proportional to the number of publications.

#### 2.3.4. Evolution by Region over Time

To study the behavior of the research field during the past decade, we analyzed the number of research publications by region over time. This analysis provided us with insights into how different regions in LAC have contributed to the development of the CSID and information-systems research field. In addition, the temporal analysis tracks the number of publications and the growth trajectory of research activities across the prioritized regions. Understanding these trends helps to identify the dynamics of research. To visualize this evolutionary phenomenon, we used a stacked bar chart where the x-axis represents the year of publication, and the y-axis represents the number of publications, with colors assigned to the main region of the origin of the article.

In this study, the regional classification was based on the cultural distribution of the Americas (Latin and Anglo-Saxon) and the geographical distribution (North, Central, and South America). For those papers that explicitly include terms such as Latin America, South America, North America, Central America, and their combinations, the classification was chosen in this way. For the papers that include analysis of one or more countries located in the regions, the category selected was the main region of analysis based on the geographical distribution framework.

#### 2.3.5. Evolution of CSID Research over Time

To analyze the evolution of CSID research over time, we first segmented the data by publication year and then categorized each publication on the basis of the specific CSID addressed. The data were visualized by using a stacked bar chart, where the x-axis represents the publication year, and the y-axis represents the number of publications, with each CSID assigned a distinctive color. We conducted a comparative analysis to understand which diseases garnered more attention over time, and we identified emerging patterns or shifts in research focus. This comprehensive analysis provided valuable insights into the dynamics of scientific attention and resource allocation within the field of CSID.

#### 2.3.6. Geographical Distribution of Research by CSID

The geographical distribution of research was studied by focusing on the distribution of CSID research in each country. We categorized research publications on the basis of the specific CSID that they addressed, and we then mapped these publications to their respective geographic locations. We use a map of LAC with pie charts for each country to represent the distribution of research on different CSIDs. The pie charts are color-coded to indicate different diseases, providing a clear visual representation of the research focus in each area. Trend analysis was performed to identify regions with high research activity for specific CSIDs, and to identify geographical patterns and hotspots. Comparative analysis helped us to understand regional specialization and its relationship to the prevalence of certain diseases in certain areas. This comprehensive geographical analysis provided insight into the regional focus and distribution of CSID research activity.

#### 2.3.7. Relationship Between Analytical Methods, CSID, and Number of Publications

We analyzed the relationship between the three variables: analytical methods, CSID, and number of publications. We chose radial graphs to show the relationships among the three variables: the number of research papers on CSID, the analytical methods used, and the diseases. These graphs organize the data into concentric circles, with the inner ring classifying one variable, and the outer rings detailing the second variable. The height of each bar is proportional to the number of articles, providing an intuitive understanding of the volume of research for each category.

By examining the density and distribution of connections, we can understand the methodological trends within the field of CSID. Comparative analysis of the variables helped us to identify methodological gaps and areas where certain techniques might be underutilized. This comprehensive analysis facilitates a deeper understanding of the methodological landscape regarding CSID research, guiding future studies towards the most effective and innovative analytical approaches.

## 3. Results

**Figure 2:**
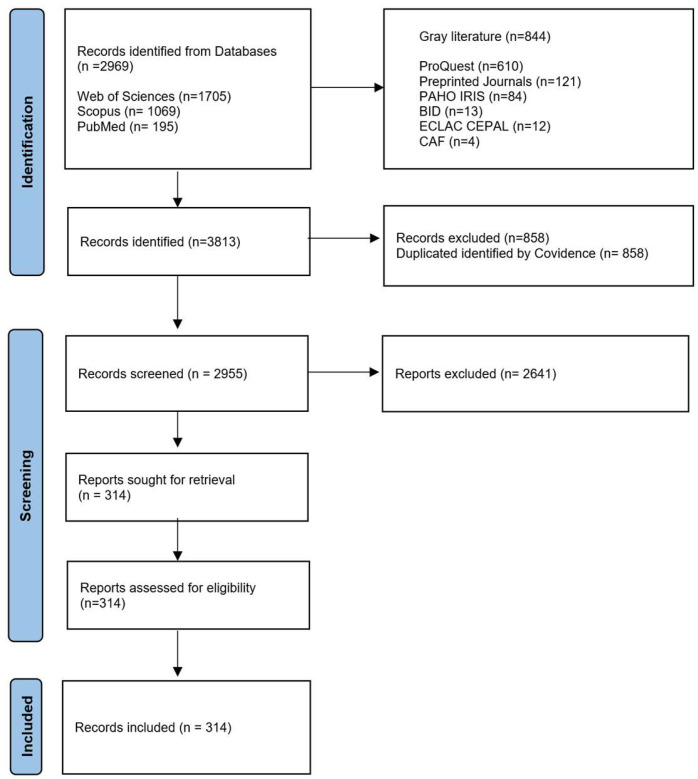
PRISMA flow diagram.

Figure 2 presents the PRISMA flowchart for the selection of documents that fulfil the inclusion criteria. A total of 2969 records were retrieved from the academic databases, and 844 records were extracted from the gray-literature databases and repositories, resulting in a total of 3813 records identified. After eliminating duplicates, 2955 records were selected for title and abstract screening. Of those, 314 articles were selected for full-text screening and were used for the bibliometric analysis.

### 3.1. Life-Cycle Analysis

**Figure 3:**
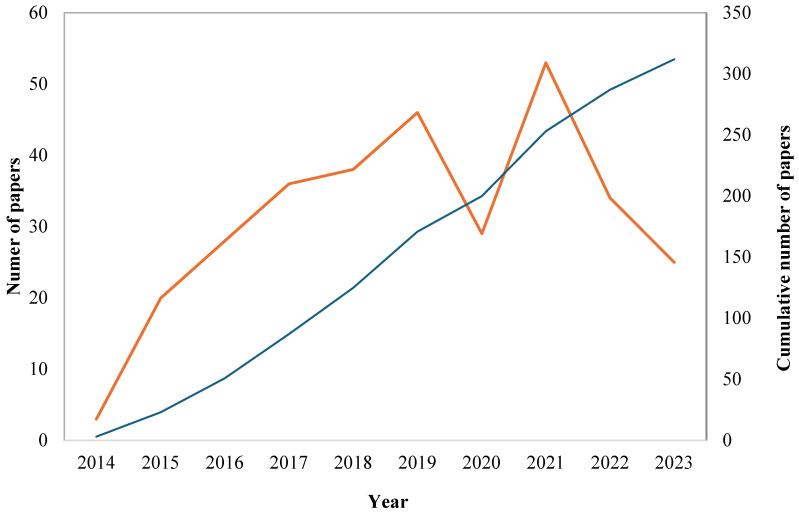
Life-cycle chart of scientific papers related to CSID and information systems. The orange line represents the number of papers in each year, and the blue line represents the cumulative number of papers.

To understand how research on information systems including CSID has progressed in the LAC region over the past decade, we performed a life-cycle analysis of scientific papers, based on the published time evolution plot (Figure 3). Research output expanded rapidly between 2014 and 2019, reflecting growing regional attention to climate–health interactions. The slower and more variable production after 2020 may relate to shifts in research funding and competing public-health priorities during the COVID-19 pandemic.

Based on the simplified analysis framework proposed by Singh [32], the evolutionary stages of a scientific field can be identified using the following phases: creation, adoption, peak, and decay. When mapped onto standard life-cycle stages, the field shows an initiation phase (2014–2015), a period of expansion (2016–2019), a publication peak (2020–2021), and lower output in the subsequent years. For the specific field examined in this study, these phases can be identified as follows.

Creation (2014–2015): This phase is characterized by the initial emergence of the field, where the number of publications begins to rise. In Figure 3, there is a noticeable increase in the number of papers, starting from 2014, indicating the creation phase.

Adoption (2016–2019): During the adoption phase, the field gains more attention, and the growth in publications is more rapid. Figure 3 shows a steady increase in the number of papers published each year from 2016 to 2019. This period represents the adoption phase, where the research community increasingly engages with the field.

Peak (2020–2021): The peak phase is when the field reaches its highest level of activity and the maximum number of publications. The peak in this case occurs in 2020 and 2021, where the number of publications is at its highest point.

Possible Decay (2022–2023): The decay phase follows the peak, characterized by a decline in the number of publications. Figure 3 shows a drop in the number of papers published from 2022 onwards, indicating a possible decay phase.

### 3.2. Geographical Distribution of Research

**Figure 4:**
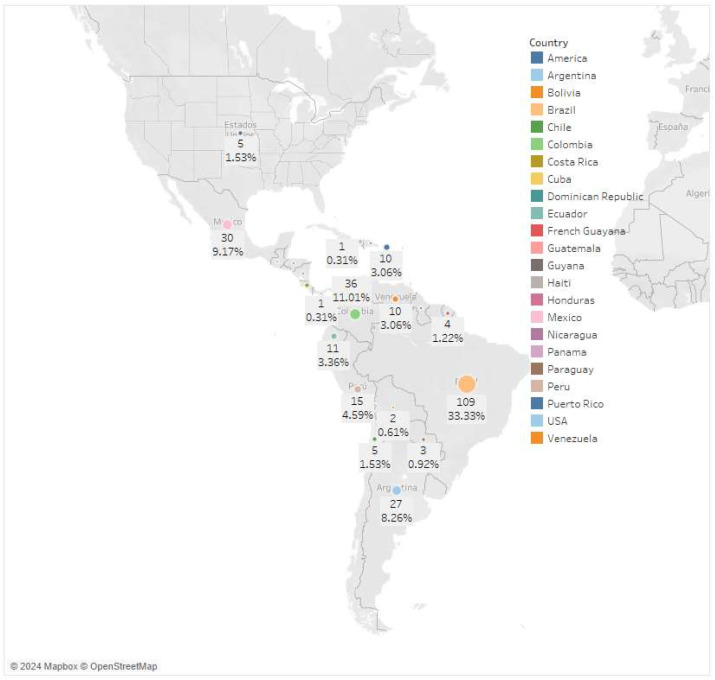
Distribution of publications by LAC countries.

Figure 4 shows the distribution of the number of documents across LAC countries.

Research output is unevenly distributed across the LAC region. Brazil accounts for the largest share of publications, contributing 109 documents (33.33%), followed by Colombia with 36 (11.01%) and Mexico with 30 (9.17%). Most other countries show substantially lower output, while 38 studies adopt a regional scope (e.g., Latin America, Central America, South America), which explains their absence as individual country entries in Figure 4.

Table 1 presents the distribution of studies grouped by region. The number of studies in South America stands out significantly, indicating a high level of research activity related to CSID and information systems in this region. North America and the Caribbean region follow, while global studies comprise the fourth portion. Central America, the Americas, the combined regions of the Americas and the Caribbean, and the combined regions of South America and the Caribbean follow, with fewer studies.

**Figure 5:**
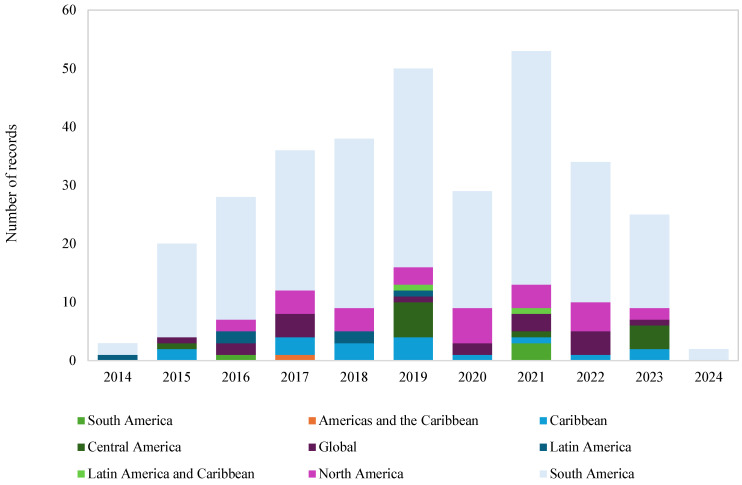
Evolution of the generation of scientific papers by regional group over time.

The temporal analysis by region (Figure 5) shows that South America, North America, and the Caribbean have been the most active regions in scientific production in recent years. South America accounts for most publications across the decade, with varying annual outputs and clear peaks in 2018 (31 documents), 2019 (34), and 2021 (40). North America and the Caribbean contribute smaller but steady volumes, generally between two and seven publications per year.

**Figure 6:**
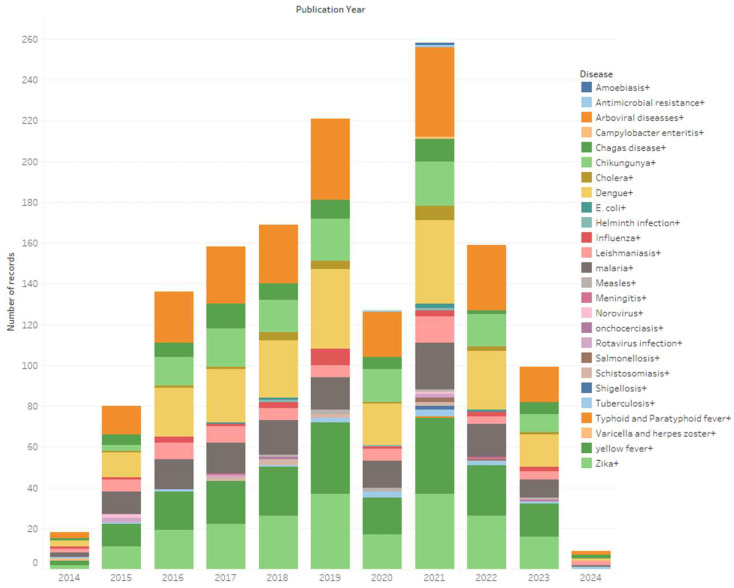
Temporal analysis of CSID.

The analysis of disease-specific research frequencies shows a predominant focus on arboviral infections, which together account for 182 studies (57.96% of the total). Within this group, dengue represents the largest share, followed by chikungunya, Zika, and yellow fever. Leishmaniasis and malaria show steady publication levels across the decade, while less frequently studied CSIDs (including onchocerciasis, schistosomiasis, varicella and herpes zoster, measles, and cholera) each contribute fewer than 1% of all documents. This distribution highlights the main areas of research emphasis within CSID and analytical methods (Figure 6).

**Figure 7:**
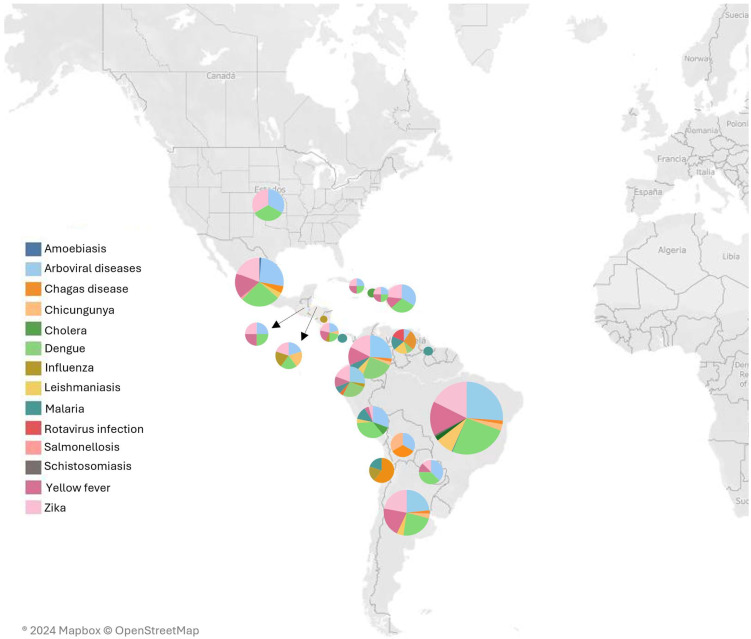
Geographical distribution of research in CSID.

Argentina, Brazil, Colombia, Costa Rica, Cuba, Ecuador, Mexico, Paraguay, Puerto Rico, and the Dominican Republic show a similar distribution of research by disease, with arboviruses, dengue, Zika, and yellow fever as the most researched diseases in these countries. Bolivia, Chile, Haiti, Guyana, Panama, and Venezuela show clear differences in the distribution of disease focus (Figure 7).

Table 2 presents the distribution of analytical methods in the included studies. Regression models, spatial analysis, and time-series analysis were the most used methods in the identified studies.

In this research field, a variety of advanced modeling techniques have been employed to understand disease dynamics and to predict future trends. Our approach highlights the distribution of research across diseases and analytical methods, identifying areas with extensive literature and potential gaps for further investigation. Analyzing the methods used over the years reveals trends in disease research. Niche modeling has been consistently popular since 2015, with peaks in 2016 (7) and 2017 (11). Time-series analysis shows a steady increase in use, peaking in 2021 (4). Regression models have also been widely used, especially in 2017 (8) and 2020 (10). Advanced statistical models and Bayesian analysis, with 12 studies, have seen growing adoption in recent years, reflecting not only increased interest in more sophisticated approaches for understanding disease dynamics and assessing risk factors, but also the expanding availability of computational resources (such as open-source statistical software and cloud-based processing) that have made these methods more accessible to researchers in the region. Neural networks and other machine learning methods have gained traction, particularly since 2018, demonstrating the advances in artificial intelligence techniques for predicting disease outbreaks. These evolving methodologies underscore the field’s shift towards more complex and data-driven approaches, enhancing the understanding of CSID transmission and informing public-health interventions. Appendix K shows the list of the most relevant articles in each group by disease and information analysis technique category.

**Figure 8:**
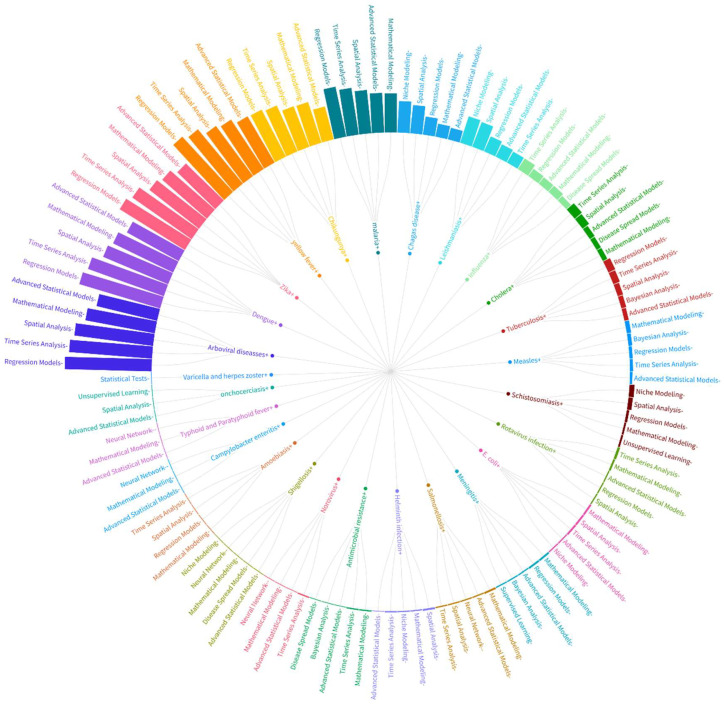
Radial network visualization illustrating analytical methods (outer ring) and the climate-sensitive infectious diseases to which they are applied (inner ring). Bar height represents the number of publications employing each method–disease pairing and lines connecting the rings indicate the direction of the association. Colors group diseases according to ecological or epidemiological similarity. The visualization summarizes the frequency and structure of method application across diseases.

**Figure 9:**
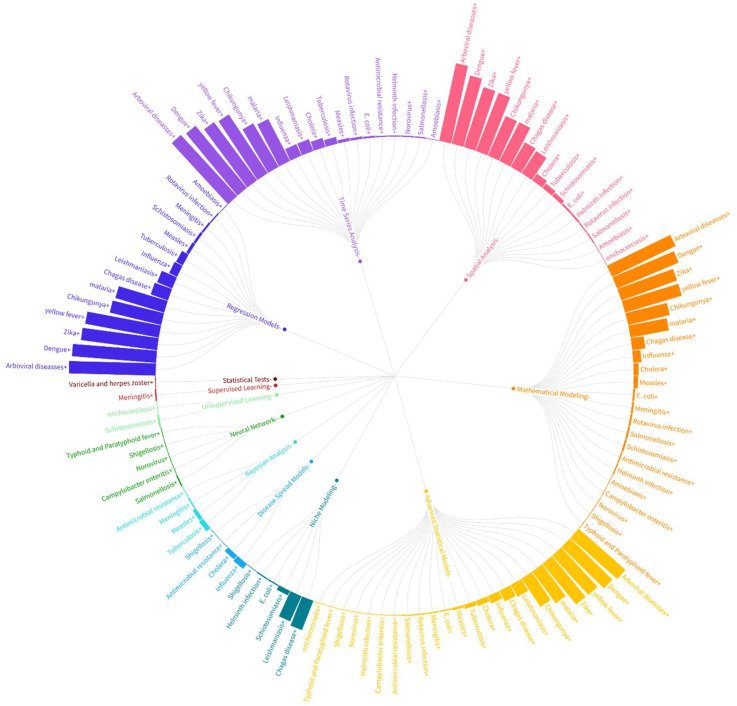
Radial network visualization showing climate-sensitive infectious diseases (outer ring) and the analytical methods used to study them (inner ring). Bar height denotes the number of publications linking each disease to a specific analytical approach. Connecting lines represent method–disease relationships derived from the full-text corpus. Colors group analytical approaches with similar modeling logic (e.g., regression, time-series, spatial, or advanced statistical methods). The visualization displays the distribution of methodological choices across the CSID spectrum.

Figure 8 and Figure 9 use radial network diagrams to summarize relationships between analytical methods and climate-sensitive infectious diseases (CSIDs). In these visualizations, bar height represents the number of publications reporting a given method–disease association. Lines connecting the outer and inner rings indicate the direction of the relationship, and colors group elements according to methodological or epidemiological similarity. This format provides a concise visual summary of the analytical emphasis across CSIDs in the region and highlights how methodological families differ in their application to specific pathogens.

Analysis of the data reveals significant insights into the most frequently used analytical techniques, and their relationships with various diseases. In Figure 8, we illustrate the connections between different statistical and computational methods and specific CSIDs, highlighting key areas of focus and methodological trends. The arboviral diseases, dengue, Zika, yellow fever, and chikungunya, follow the same order in the top five of the analytical methods used. A similar trend is seen in malaria research, with a shift in the fourth and fifth most employed techniques, which are advanced statistical models and mathematical modeling. The less-studied diseases are varicella and herpes zoster, onchocerciasis, typhoid and paratyphoid fever, campylobacter enteritis, amoebiasis, shigellosis, norovirus, antimicrobial resistance, helminth infection, and salmonellosis.

As Figure 9 shows, regression models are the most-used methods in the study of CSID. Other methods include time-series analysis, spatial analysis, mathematical modeling, advanced statistical models, niche modeling, disease-spread models, Bayesian analysis, neural networks, and unsupervised learning. The two least-used methods are statistical tests and supervised learning. Advanced statistical models are the most important in terms of methods applied to a wide range of diseases, being used in the study of 23 diseases. This is followed by mathematical modeling, time-series analysis, spatial analysis, and regression models, which are applied to 22, 18, 17, and 15 diseases, respectively.

**Figure 10:**
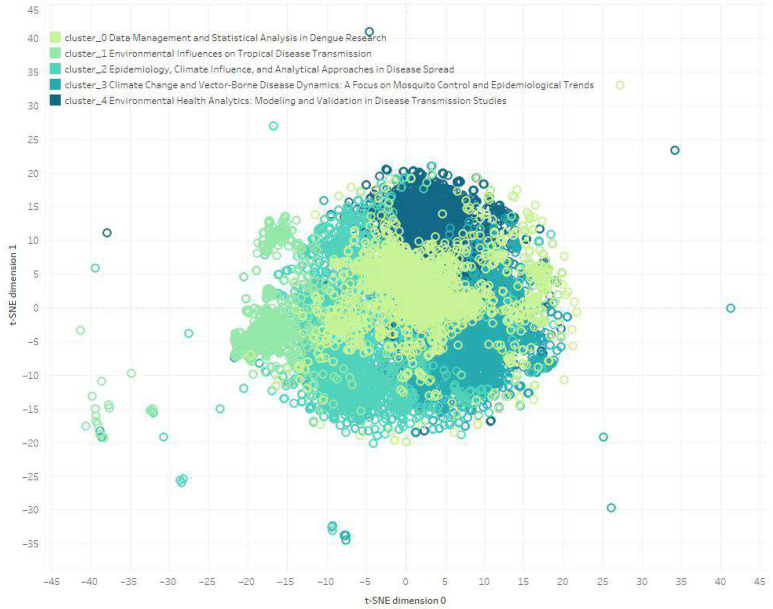
t-SNE plot of sentence-level embeddings grouped into five thematic clusters. Each point represents an individual sentence extracted from the full-text corpus, and colors denote cluster assignments. Labels summarize dominant themes in each cluster: (0) data management and dengue-focused statistical analysis, (1) environmental influences on transmission, (2) epidemiology and climate-related analytical approaches, (3) climate change and vector-borne disease dynamics, and (4) environmental health modeling and validation. The t-SNE projection is used only to visualize thematic similarity among sentences and does not reflect geographic or temporal distances.

Through the application of embeddings analysis and t-SNE dimension reduction, we have identified five distinct clusters within the scientific literature (clusters 0–4). These clusters represent thematic groupings that encapsulate the relationship between CSIDs, analytical methods, and climate variables. Clusters enable us to understand the main trends in the scientific topic. Figure 10 visually represents these clusters, distinguished by colors.

Cluster 0 focuses on dengue-fever research, emphasizing data-management practices and statistical analysis techniques. This cluster discusses the collection, management, and analysis of data related to dengue incidence, vector distribution, and the influence of climatic variables such as temperature, humidity, and precipitation on disease spread. Methods include regression analyses, time-series analysis, and machine learning models like decision trees and neural networks to predict dengue outbreaks and understand transmission mechanisms.

Cluster 1 examines the relationship between environmental factors and the transmission of diseases, particularly the effects of climate change, deforestation, and urbanization on disease vectors and reservoirs. It emphasizes the impacts of temperature, precipitation, humidity, and sea-level rise on the spread of disease. Methods used include correlation and regression analyses, machine learning models, and mathematical models to simulate disease transmission under various environmental scenarios.

Cluster 2 delves into the interplay between CSID and climate variables, covering diseases such as malaria, dengue, and Zika. It highlights the importance of temperature, humidity, and rainfall in disease-transmission cycles. Methods involve regression models, time-series analysis, spatial statistics, and machine learning techniques such as predictive modeling and neural networks to understand and predict disease outbreaks.

Cluster 3 investigates the connections between climate variables and vector-borne diseases, with a focus on mosquito-borne illnesses. It analyzes how temperature, precipitation, and humidity influence mosquito life-cycles and disease spread. Statistical methods, machine learning models, and mathematical modeling techniques, including compartmental models and ecological niche models, are employed to understand and predict disease dynamics in different climatic scenarios.

Cluster 4 explores the application of statistical and AI methodologies in modeling the interactions between environmental variables and disease transmission. It covers data validation and the impact of environmental changes such as land use and urbanization on public health. Methods include linear and nonlinear modeling, regression analysis, time-series analysis, and machine learning techniques such as random forests and neural networks to predict disease outbreaks and assess the impact of environmental factors.

## 4. Discussion

The main purpose of this study was to examine how research on analytical models applied to climate-sensitive infectious diseases (CSIDs) has evolved in Latin America and the Caribbean over the last decade. Our results show that arboviral infections, particularly dengue, Zika, chikungunya, and yellow fever, dominate the regional literature. This pattern reflects the established burden of mosquito-borne diseases in tropical and subtropical settings [33,34] and the continuing need for analytical models capable of supporting risk assessment and operational decision making for this diseases. Conversely, conditions such as cystic echinococcosis, shigellosis, campylobacter enteritis, typhoid and paratyphoid fevers, pneumococcal disease, and varicella and herpes zoster appear only sporadically in the research. These gaps suggest that some CSIDs receive proportionally less analytical attention, possibly due to lower perceived urgency, limited data availability, or narrower national research agendas.

The life-cycle analysis suggests a sequence resembling initiation, growth, and peak activity within the study period, although the relatively short 10-year window restricts certainty regarding long-term dynamics. The observed decline in publications after 2021 should be interpreted cautiously, as short-term fluctuations, particularly disruptions related to the COVID-19 pandemic [35], may influence output. Importantly, this decade corresponds to a broader rise in the application of analytical and computational approaches in public health [36], as well as growing scientific interest in climate–health interactions [7,37]. Extending the temporal window in future studies would allow for a clearer assessment of whether the field is stabilizing, diversifying, or shifting toward new methodological paradigms.

Regionally, Brazil stands out as the dominant contributor to CSID analytical modeling research. This is consistent with its scientific production capacity in public health [38], sustained investment in research and development [39], and high incidence of arboviral diseases [40].

Countries like Argentina, Brazil, Colombia, Costa Rica, Cuba, Ecuador, Mexico, Paraguay, Puerto Rico, and the Dominican Republic show similar research trends, focusing primarily on arboviral diseases such as dengue, Zika, and yellow fever. In contrast, countries such as Bolivia, Guyana, Haiti, Panama, and Venezuela present more heterogeneous or less concentrated analytical activity, which may reflect differences in research capacity, funding stability, and disease reporting systems. Chile is a distinctive case, characterized as having a strong research and development system, based on its position in the Global Innovation Index—GII [41], but it presents low incidences of the most common CSIDs in the study, such as dengue [42], Zika [43], and chikungunya [44].

Our findings align with those of López et at [11], who examined scientific production related to climate variability and CSID. Their observation that Brazil, Colombia, and Argentina led the region is consistent with our results and reinforces the continuity of research priorities across decades. Likewise, the prominence of dengue and leishmaniasis in their analysis corresponds with the disease patterns identified in our dataset.

We find that regression models, time-series analysis, and spatial analysis are the top analytical methods used to study CSID in the region. In LAC-focused research, these three methodological families dominate the literature, appearing far more frequently than complex mechanistic or machine learning models. This finding is further supported by studies that have reviewed the literature and revealed that the majority of models used for outbreak prediction and transmission risk assessment in arboviral diseases were built using conventional regression techniques [45,46]. Regression models, particularly generalized linear models such as Poisson regressions, are widely used in LAC CSID studies due to their accessibility, ease of implementation, and interpretability. Their popularity stems from their capacity to quantify relationships between variables, clarify complex interactions in disease dynamics [45], handle over-dispersion in epidemiological data, and produce estimates that are both interpretable and useful for informing public health responses. Studies have shown that the time-series technique is particularly effective in predicting the highly auto-correlated nature of dengue infection [46]. While the time-series method is a more robust modeling technique, its use is less common than that of regression models. Geographic Information System (GIS)-based spatial analysis is extensively used to visualize and investigate the geographic distribution of disease outbreaks. These spatial techniques have been instrumental in identifying ecological and climatic drivers of transmission, enabling more targeted public health interventions and enhancing the understanding of spatial dynamics in disease ecology.

Techniques such as Bayesian models, niche modeling, and machine learning (e.g., supervised and unsupervised learning) tend to have higher technical requirements. Bayesian methods are generally considered more complex, due to their probabilistic framework and the incorporation of prior knowledge or beliefs into the analysis [47,48]. On the other hand, niche modeling, which focuses on predicting species distributions based on environmental variables, may involve sophisticated statistical and computational techniques but is often more straightforward compared with Bayesian methods in spatial epidemiology [49]. Finally, the complexity of machine learning and neural networks can create barriers to their widespread implementation. Significant computational resources, specialized expertise, and robust data infrastructures are required in order to utilize these methods effectively.

A practical implication of these findings is that the analytical approaches most commonly used in the region, such as generalized linear models, time-series forecasting, and spatial analysis, already provide actionable support for climate-related disease monitoring and early-warning workflows. For example, generalized linear models combined with entomological monitoring have been used to predict Aedes aegypti infestation trends under varying meteorological conditions [50], and drought- and temperature-driven ecological responses of vectors have been characterized for triatomines and mosquito species [51,52]. Earth-observation-based modeling also demonstrates how remote-sensing data can enhance dengue early-warning systems [53], and time-series approaches such as SARIMA have been applied to forecast visceral leishmaniasis incidence at subnational scales [54]. Spatial epidemiological models further contribute to identifying climatic drivers of malaria transmission in cross-border regions [55]. Only a limited number of studies have implemented more advanced analytical frameworks, including Bayesian hierarchical spatiotemporal models for dengue prediction [56]. Their scarcity in the regional literature reflects persistent constraints such as the limited availability of long and high-quality climate–health time series, heterogeneous data integration across surveillance systems, restricted computational capacity within public-health institutions, and disciplinary gaps between model developers and applied disease-control programs. As a result, regression models, time-series analyses, and spatial approaches continue to dominate because they require fewer resources and can be more readily incorporated into operational public-health decision making.

These results carry important implications for both research and public health planning in LAC. The continued reliance on foundational methods reflects a pragmatic adaptation to prevailing data and resource constraints. Strengthening public health information systems by improving data availability, standardization, and cross-sectoral interoperability could enable the wider adoption of advanced modeling tools, such as machine learning and Bayesian methods, thereby enhancing forecasting accuracy and risk stratification. The strong research emphasis on arboviral diseases aligns with their regional disease burden but underscores the need to broaden analytical focus to other CSIDs, including water-borne and parasitic infections, which remain underexamined. Finally, the observed growth in studies integrating climate data into disease surveillance supports the development of locally adapted early-warning systems that can deliver timely, location-specific insights to guide preparedness, allocate resources efficiently, and inform climate-resilient public health strategies.

Methodologically, this study contributes to CSID research by integrating author-led filtering with full-text NLP workflows, enabling a more granular characterization of analytical methods. However, some limitations should be acknowledged. The regional scope restricts generalizability beyond the LAC context. In addition, although the NLP pipeline improves classification accuracy compared with title/abstract-only approaches, it remains dependent on keyword frequency and may miss context-specific nuances. Finally, because the t-SNE clustering was conducted at the sentence level and the workflow did not retain identifiers linking each sentence to its source article, it is not possible to determine the number or identity of articles represented in each thematic cluster.

Future research could expand the geographic and temporal scope, integrate additional metadata (such as collaboration networks or funding flows), and incorporate complementary methodologies such as full scoping reviews or validated machine learning classification tools. Doing so would deepen our understanding of analytical trends, strengthen cross-country comparisons, and support the development of climate-informed public-health strategies.

## 5. Conclusions

This article uses a bibliometric analysis to evaluate the evolution and distribution of research on CSIDs in Latin America and the Caribbean. It uses bibliometric and text-mining techniques to analyze publication trends, research hotspots and methodological development between 2015 and 2024. The findings reveal that Brazil is the country leading research in CSID, focusing mainly on dengue, Zika and chikungunya. Regression models, time-series and spatial analysis are the main research methods used in the region. Although research in CSIDs has grown in the region, there are still essential inequalities in the number and type of research produced, and the countries involved in designing and conducting the research. It is fundamental to strengthen the region’s capabilities and integrate analytical models into public health to anticipate the impact of climate change.

## Figures and Tables

**Table 1:** Distribution of studies by region.

Region	Number of Studies *	Percentage (%) **
South America	242	73.78%
North America	32	9.76%
Caribbean	17	5.18%
Global	18	5.49%
Central America	12	3.66%
Americas	4	1.22%
Americas and Caribbean (combined)	2	0.61%
South America and Caribbean (combined)	1	0.30%

* The sum of the total is not 314 but 328, as 14 articles have an interregional scope. ** The percentage was calculated by taking 328 as the total.

**Table 2:** Distribution of analytical methods used in studies.

Analytical Method	Percentage (%) *
Regression models	12.7%
Spatial analysis	12.1%
Time-series analysis	11.8%
Mathematical modeling	10.2%
Advanced statistical models	9.4%
Niche modeling	8.8%
Bayesian analysis	6.2%
Unsupervised learning	6.0%
Neural networks	5.9%
Statistical tests	5.1%
Disease-spread models	4.9%
Specific models	2.9%
Supervised learning	2.4%
Other models	0.7%

(*) The percentage is calculated based on the total number of mentions across all articles, as some documents employ more than one method.

## Data Availability

Data sharing is not applicable to this article as no new data were created or analyzed in this study.

## Appendix A. Search Strategies Scopus

**Characteristic**	**Report**
Type of search	New
Database	SCOPUS
Platform	SCOPUS
Search date	13 December 2023
Date range for the search	2015–Current
Search strategy	(TITLE-ABS-KEY(vector-borne OR “vector borne” OR “vector transmission” OR mosquito-borne OR aedes-borne OR “Aedes aegypti” OR aedes OR leishmania* OR “Chaga* disease” OR trypanosom* OR chaga* OR dengue* OR “Breakbone Fever” OR “yellow fever*” OR “fiebre amarilla” OR malaria OR plasmodium OR “Marsh fever” OR zikv OR zika OR chikungunya OR waterborne OR “water*borne” OR foodborne OR “food*borne” OR “feed*borne” OR shigell* OR “escherichia coli” OR e.coli OR “e coli” OR “e*coli” OR campylobacter OR salmonell* OR typhoid OR paratyphoid OR amoebiasis OR “Amebic Dysentery” OR dysenteries OR ameb* OR soilborne OR “soil*borne” OR tuberculos* OR “Kochs Disease” OR influenza OR “Antimicrobial resistanc*” OR rotavirus OR (“Climate sensitive” W/5 disease*) OR (“Climate sensitive” W/5 infect*) OR (“Climate sensitive” W/5 illness*) OR (Climate-sensitive W/5 disease*) OR (Climate-sensitive W/5 infect*) OR (Climate-sensitive W/5 illness*) OR (Climat* W/5 disease*) OR (Climat* W/5 infect*) OR (Climat* W/5 illness*) OR (Climat* W/5 sensitiv*) OR csid* OR csd* OR “Entamoeba histolytica” OR pneumococ* OR streptococc* OR varicella-zoster OR herpesvir* OR herpes OR varicellae OR chickenpox OR “VZ Virus” OR measles OR rubeola OR morbilli* OR cholera OR norovirus OR onchoce* OR echinococ* OR helminth* OR “Ascaris lumbricoide*” OR “Trichuris trichiura” OR hookworms OR schistosom* OR meningi* OR Tickborne OR Tick*borne OR “tick borne” OR Cysticerc* OR Cisticercos* OR coenur* OR epidemic* OR pandemic*) AND TITLE-ABS-KEY((management* W/5 informa*) OR (system* W/5 informa*) OR (integration W/5 system*) OR (model* W/5 system*) OR (spatiotemporal W/3 model*) OR (Health* W/5 Informa*) OR (data W/3 system*) OR “Decision Support System” OR “computer-based model*” OR (digital* W/3 tool*) OR (internet* W/5 data) OR GIS* OR “geographical information system*” OR (Information W/5 System*) OR (geo* W/3 system*) OR forecast* OR “geos* data” OR (epidemiolog* W/3 assessment) OR “early warning system*” OR EWS OR “Early Warning and Response System*” OR EWARS OR “regional data shar*” OR “big data” OR bigdata OR “machine learning” OR “artificial intelligence” OR AI OR “deep learning” OR “data mining” OR “predic* analys*” OR modelling OR “neural network” OR software* OR simulation* OR (dynamic W/3 anal*) OR (multicriter* W/5 evaluation*)) AND TITLE-ABS-KEY(climat* OR weather OR meteorolog* OR “atmospheric condition*” OR “Climate change” OR “changing climate” OR “climate-informed” OR “global change” OR “cambio clim*tico” OR (Clima* W/5 epidemiolog*) OR (Clima* W/5 modelling) OR (clima* W/5 data) OR (clima* W/5 system*) OR (clima* W/5 service*) OR (clima* W/5 partnership) OR (clima* W/5 health*) OR (clima* W/5 forecast*) OR (clima* W/5 informa*) OR “Extreme weather event” OR Cyclone OR “Extreme weather” OR Flood* OR “Heat wave” OR Hurricane OR Storm OR Moisture OR Altitude OR “Dew point” OR Humid* OR “Saturation deficit” OR “Soil moisture” OR Oscillation* OR “La Ni*a” OR “El Ni*o” OR “North Atlantic Oscillation” OR “Pacific Decadal Oscillation” OR ENSO OR ENOS OR “Partic* matter” OR Dust* OR Turbidity OR Rain* OR Drought OR “Heavy rain” OR “High rainfall” OR Monsoon OR Precipitation OR “Annual rainfall” OR Runoff OR Wet* OR temperature* OR Freezing OR “Warm years” OR Warm* OR Winter OR Wind* OR NDVI OR Salinity OR deforestation OR drought OR adaptation OR variability) AND TITLE-ABS(“Latin America” OR “South America” OR “Central America” OR “Caribbean” OR “Am*rica Latina” OR latin* OR “Am*rica do Sul” OR “Am*rica Central” OR Caribe OR Aruba OR Argentina OR “Antigua y Barbuda” OR “Ant*gua * Barbuda” OR Bahamas OR Belize OR Bol*via OR Bra*il OR Barbados OR Chile OR Colombia OR “Costa Rica” OR Cuba OR “Ilhas Cayman” OR “Cayman Islands” OR Dominica* OR E*uador OR Granada OR Grenada OR Guatemala OR Guiana OR Guyana OR Honduras OR Haiti OR Jamaica OR M*xico OR Nicar*gua OR Panam* OR Peru OR “P*rto Rico” OR Paraguai OR Paraguay OR “El Salvador” OR Suriname OR “Saint Maarten” OR “Saint Maarten” OR “Ilha de Turks e Caicos” OR “Turks and Caicos” OR “Trinidad * Tobago” OR Urugua* OR “S*o Vicente e Granadinas” OR “St. Vincent and the Grenadines” OR Venezuela OR “Ilhas Virgens” OR “Virgin Islands”) AND NOT TITLE (COVID* OR SARS*)) AND PUBYEAR > 2014 AND PUBYEAR < 2025
References	1069

## Appendix B. Search Strategies in Web of Sciences

**Characteristic**	**Report**
Type of search	New
Database	Web of Science
Platform	Web of Science
Search date	13 December 2023
Date range for the search	2015–Current
Search strategy	((((TS = (vector-borne OR “vector borne” OR “vector transmission” OR “mosquito-borne” OR “Aedes aegypti” OR aedes OR leishmania* OR “Chagas disease” OR trypanosom* OR chagas OR dengue* OR “Breakbone Fever” OR “yellow fever*” OR “fiebre amarilla” OR malaria OR plasmodium OR “Marsh fever” OR zikv OR zika OR chikungunya OR waterborne OR “water*borne” OR foodborne OR “food*borne” OR “feed*borne” OR shigell* OR “escherichia coli” OR e.coli OR “e coli” OR “e*coli” OR campylobacter OR salmonell* OR typhoid OR paratyphoid OR amoebiasis OR “Amebic Dysentery” OR dysenteriae OR ameb* OR soilborne OR “soil*borne” OR tuberculos* OR “Koch’s Disease” OR influenza OR “Antimicrobial resistanc*” OR rotavirus OR (“Climate sensitive” NEAR/5 disease*) OR (“Climate sensitive” NEAR/5 infect*) OR (“Climate sensitive” NEAR/5 illness*) OR (Climate-sensitive NEAR/5 disease*) OR (Climate-sensitive NEAR/5 infect*) OR (Climate-sensitive NEAR/5 illness*) OR (Climat* NEAR/5 disease*) OR (Climat* NEAR/5 infect*) OR (Climat* NEAR/5 illness*) OR (Climat* NEAR/5 sensitiv*) OR csid* OR csd* OR “Entamoeba histolytica” OR pneumococ* OR streptococc* OR varicella-zoster OR herpesvir* OR herpes OR varicella OR chickenpox OR “VZ Virus” OR measles OR rubeola OR morbilli* OR cholera OR norovirus OR onchoce* OR echinococ* OR helminth* OR “Ascaris lumbricoide*” OR “Trichuris trichiura” OR hookworms OR schistosom* OR meningi* OR Tickborne OR Tick*borne OR “tick borne” OR Cysticerc* OR Cisticercos* OR coenur* OR epidemic* OR pandemic*)) AND TS = ((management* NEAR/5 informa*) OR (system* NEAR/5 informa*) OR (integration NEAR/5 system*) OR (model* NEAR/5 system*) OR (spatiotemporal NEAR/3 model*) OR (Health* NEAR/5 Informa*) OR (data NEAR/3 system*) OR “Decision Support System” OR “computer-based model*” OR (digital* NEAR/3 tool*) OR (internet* NEAR/5 data) OR GIS* OR “geographical information system*” OR (Information NEAR/5 System*) OR (geo* NEAR/3 system*) OR forecast* OR “geos* data” OR (epidemiolog* NEAR/3 assessment) OR “early warning system*” OR EWS OR “Early Warning and Response System*” OR ewart OR “regional data shar*” OR “big data” OR bigdata OR “machine learning” OR “artificial intelligence” OR AI OR “deep learning” OR “data mining” OR “predic* analys*” OR modelling OR “neural network” OR software* OR simulation* OR (dynamic NEAR/3 anal*) OR (multicriter* NEAR/5 evaluation*))) AND TS = (climat* OR weather OR meteorolog* OR “atmospheric condition*” OR “Climate change” OR “changing climate” OR “climate-informed” OR “global change” OR “cambio clim*tico” OR (Clima* W/5 epidemiolog*) OR (Clima* W/5 modelling) OR (clima* W/5 data) OR (clima* W/5 system*) OR (clima* W/5 service*) OR (clima* W/5 partnership) OR (clima* W/5 health*) OR (clima* W/5 forecast*) OR (clima* W/5 informa*) OR “Extreme weather event” OR Cyclone OR “Extreme weather” OR Flood* OR “Heat wave” OR Hurricane OR Storm OR Moisture OR Altitude OR “Dew point” OR Humid* OR “Saturation deficit” OR “Soil moisture” OR Oscillation* OR “La Ni*a” OR “El Ni*o” OR “North Atlantic Oscillation” OR “Pacific Decadal Oscillation” OR ENSO OR ENOS OR “Partic* matter” OR Dust* OR Turbidity OR Rain* OR Drought OR “Heavy rain” OR “High rainfall” OR Monsoon OR Precipitation OR “Annual rainfall” OR Runoff OR Wet* OR temperature* OR Freezing OR “Warm years” OR Warm* OR Winter OR Wind* OR NDVI OR Salinity OR deforestation OR drought OR adaptation OR variability)) AND TS = (“Latin America” OR “South America” OR “Central America” OR “Caribbean” OR “Am*rica Latina” OR latin* OR “Am*rica do Sul” OR “Am*rica Central” OR Caribe OR Aruba OR Argentina OR “Antigua y Barbuda” OR “Ant*gua * Barbuda” OR Bahamas OR Belize OR Bol*via OR Bra*il OR Barbados OR Chile OR Colombia OR “Costa Rica” OR Cuba OR “Ilhas Cayman” OR “Cayman Islands” OR Dominica* OR E*uador OR Granada OR Grenada OR Guatemala OR Guiana OR Guyana OR Honduras OR Haiti OR Jamaica OR M*xico OR Nicar*gua OR Panam* OR Peru OR “P*rto Rico” OR Paraguai OR Paraguay OR “El Salvador” OR Suriname OR “Saint Maarten” OR “Saint Maarten” OR “Ilha de Turks e Caicos” OR “Turks and Caicos” OR “Trinidad * Tobago” OR Urugua* OR “S*o Vicente e Granadinas” OR “St. Vincent and the Grenadines” OR Venezuela OR “Ilhas Virgens” OR “Virgin Islands”)) NOT TI = (covid* OR sars*) [2015–2024]
References	1705

## Appendix C. Search Strategies Other Databases

**Characteristic**	**Report**
Type of search	New
Database	LILACS, MULTIMEDIA, PAHOIRIS, CUMED, VETINDEX, SES-SP, BINACIS, WPRIM, ARGMSAL, BBO and INDEXPSI
Platform	Biblioteca Virtual en Salud (BVS)
Search date	13 December 2023
Date range for the search	2015–Current
Search strategy	(((vector-borne OR “vector borne” OR “vector transmission” OR mosquito-borne OR aedes-borne OR “Aedes aegypti” OR aedes OR leishmania* OR “Chaga* disease” OR trypanosom* OR chaga* OR dengue* OR “Breakbone Fever” OR “yellow fever*” OR “fiebre amarilla” OR malaria OR plasmodium OR “Marsh fever” OR zikv OR zika OR chikungunya OR waterborne OR “water*borne” OR foodborne OR “food*borne” OR “feed*borne” OR shigell* OR “escherichia coli” OR e.coli OR “e coli” OR “e*coli” OR campylobacter OR salmonell* OR typhoid OR paratyphoid OR amoebiasis OR “Amebic Dysentery” OR dysenteries OR ameb* OR soilborne OR “soil*borne” OR tuberculos* OR “Kochs Disease” OR influenza OR “Antimicrobial resistanc*” OR rotavirus OR (“Climate sensitive disease*”) OR (“Climate sensitive infect*”) OR (“Climate sensitive illness*”) OR (“Climate-sensitive disease*”) OR (“Climate-sensitive infect*”) OR (“Climate-sensitive illness*”) OR (“Climat* disease*”) OR (“Climat* infect*”) OR (“Climat* illness*”) OR (“Climat* sensitiv*”) OR “Entamoeba histolytica” OR pneumococ* OR streptococc* OR varicella-zoster OR herpesvir* OR herpes OR varicellae OR chickenpox OR “VZ Virus” OR measles OR rubeola OR morbilli* OR cholera OR norovirus OR onchoce* OR echinococ* OR helminth* OR “Ascaris lumbricoide*” OR “Trichuris trichiura” OR hookworms OR schistosom* OR meningi* OR tickborne OR tick*borne OR “tick borne” OR cysticerc* OR cisticercos* OR coenur*)) AND (ab:(((management* informa*) OR (information system*) OR (integration system*) OR (model* system*) OR (spatiotemporal model*) OR (health* informa*) OR (data system*) OR “Decision Support System” OR “computer-based model*” OR (digital* tool*) OR (internet* data) OR gis* OR “geographical information system*” OR (geo* system*) OR forecast* OR “geos* data” OR (epidemiolog* assessment) OR “early warning system*” OR ews OR “Early Warning and Response System*” OR ewart OR “regional data shar*” OR “big data” OR bigdata OR “machine learning” OR “artificial intelligence” OR ai OR “deep learning” OR “data mining” OR “predic* analys*” OR modelling OR “neural network” OR software* OR simulation* OR (dynamic anal*) OR (multicriter* evaluation*)))) AND ((climat* OR weather OR “meteorolog*” OR “atmospheric condition*” OR “Climate change” OR “changing climate” OR “global change” OR “cambio clim*tico” OR “Extreme weather event” OR moisture OR altitude OR “Dew point” OR humid* OR “Saturation deficit” OR “Soil moisture” OR oscillation* OR “La Ni*a*” OR “El Ni*o*” OR “North Atlantic Oscillation” OR “Pacific Decadal Oscillation” OR enso OR enos OR “Partic* matter” OR rain* OR drought OR monsoon OR precipitation OR “Warm years” OR warm* OR winter OR wind* OR ndvi OR deforestation)) AND ((“Latin America” OR “South America” OR “Central America” OR “Caribbean” OR “Am*rica Latina” OR latin* OR “Am*rica do Sul” OR “Am*rica Central” OR caribe OR aruba OR argentina OR “Antigua y Barbuda” OR “Ant*gua * Barbuda” OR bahamas OR belize OR bol*via OR bra*il OR barbados OR chile OR colombia OR “Costa Rica” OR cuba OR “Ilhas Cayman” OR “Cayman Islands” OR dominica* OR e*uador OR granada OR grenada OR guatemala OR guiana OR guyana OR honduras OR haiti OR jamaica OR m*xico OR nicar*gua OR panam* OR peru OR “P*rto Rico” OR paraguai OR paraguay OR “El Salvador” OR suriname OR “Saint Maarten” OR “Saint Maarten” OR “Ilha de Turks e Caicos” OR “Turks and Caicos” OR “Trinidad * Tobago” OR urugua* OR “S*o Vicente e Granadinas” OR “St. Vincent and the Grenadines” OR venezuela OR “Ilhas Virgens” OR “Virgin Islands”))) AND (db:(“LILACS” OR “MULTIMEDIA” OR “PAHOIRIS” OR “CUMED” OR “VETINDEX” OR “SES-SP” OR “BINACIS” OR “WPRIM” OR “ARGMSAL” OR “BBO” OR “INDEXPSI”)) AND (year_cluster: [2015 TO 2024])
References	121

## Appendix D. Search Strategies PubMed

**Characteristic**	**Report**
Type of search	New
Database	PubMed
Platform	PubMed
Search date	13 December 2023
Date range for the search	2015–Current
Search strategy	(((((“vector borne”[Title/Abstract] OR “vector transmission”[Title/Abstract] OR “mosquito-borne”[Title/Abstract] OR “Aedes aegypti”[Title/Abstract] OR aedes[Title/Abstract] OR leishmania*[Title/Abstract] OR “Chagas disease”[Title/Abstract] OR trypanosom*[Title/Abstract] OR chagas[Title/Abstract] OR dengue*[Title/Abstract] OR “Breakbone Fever”[Title/Abstract] OR “yellow fever*”[Title/Abstract] OR “fiebre amarilla”[Title/Abstract] OR malaria[Title/Abstract] OR plasmodium[Title/Abstract] OR “Marsh fever”[Title/Abstract] OR zikv[Title/Abstract] OR zika[Title/Abstract] OR chikungunya[Title/Abstract] OR waterborne[Title/Abstract] OR “water*borne”[Title/Abstract] OR foodborne[Title/Abstract] OR “food*borne”[Title/Abstract] OR “feed*borne”[Title/Abstract] OR shigell*[Title/Abstract] OR “escherichia coli”[Title/Abstract] OR e.coli[Title/Abstract] OR “e coli”[Title/Abstract] OR “e*coli”[Title/Abstract] OR campylobacter[Title/Abstract] OR salmonell*[Title/Abstract] OR typhoid[Title/Abstract] OR paratyphoid[Title/Abstract] OR amoebiasis[Title/Abstract] OR “Amebic Dysentery”[Title/Abstract] OR dysenteries[Title/Abstract] OR ameb*[Title/Abstract] OR soilborne[Title/Abstract] OR “soil*borne”[Title/Abstract] OR tuberculos*[Title/Abstract] OR “Koch’s Disease”[Title/Abstract] OR influenza[Title/Abstract] OR “Antimicrobial resistanc*”[Title/Abstract] OR rotavirus[Title/Abstract] OR “Climate sensitive disease*”[Title/Abstract] OR “Climate sensitive infect*”[Title/Abstract] OR “Climate sensitive illness*”[Title/Abstract] OR “Climate-sensitive disease*”[Title/Abstract] OR “Climate-sensitive infect*”[Title/Abstract] OR “Climate-sensitive illness*”[Title/Abstract] OR “Climate disease*”[Title/Abstract] OR “Climate infect*”[Title/Abstract] OR “Climate illness*”[Title/Abstract] OR “Climate sensitiv*”[Title/Abstract] OR csid*[Title/Abstract] OR csd*[Title/Abstract] OR “Entamoeba histolytica”[Title/Abstract] OR pneumococ*[Title/Abstract] OR streptococc*[Title/Abstract] OR varicella-zoster[Title/Abstract] OR herpesvir*[Title/Abstract] OR herpes[Title/Abstract] OR varicellae[Title/Abstract] OR chickenpox[Title/Abstract] OR “VZ Virus”[Title/Abstract] OR measles[Title/Abstract] OR rubeola[Title/Abstract] OR morbilli*[Title/Abstract] OR cholera[Title/Abstract] OR norovirus[Title/Abstract] OR onchoce*[Title/Abstract] OR echinococ*[Title/Abstract] OR helminth*[Title/Abstract] OR “Ascaris lumbricoide*”[Title/Abstract] OR “Trichuris trichiura”[Title/Abstract] OR hookworms[Title/Abstract] OR schistosom*[Title/Abstract] OR meningi*[Title/Abstract] OR Tickborne[Title/Abstract] OR Tick*borne[Title/Abstract] OR “tick borne”[Title/Abstract] OR Cysticerc*[Title/Abstract] OR Cisticercos*[Title/Abstract] OR coenur*[Title/Abstract] OR epidemic*[Title/Abstract] OR pandemic*[Title/Abstract])) AND ((“management* informa*” [Title/Abstract] OR “information system*” [Title/Abstract] OR “integration system*” [Title/Abstract] OR “model* system*” [Title/Abstract] OR “spatiotemporal model*” [Title/Abstract] OR “Health* Informa*” [Title/Abstract] OR “data system*” [Title/Abstract] OR “Decision Support System” [Title/Abstract] OR “computer-based model*” [Title/Abstract] OR “digital* tool*” [Title/Abstract] OR “internet* data” [Title/Abstract] OR “GIS*” [Title/Abstract] OR “geographical information system*” [Title/Abstract] OR “geo* system*” [Title/Abstract] OR “forecast*” [Title/Abstract] OR “geos* data” [Title/Abstract] OR “epidemiolog* assessment” [Title/Abstract] OR “early warning system*” [Title/Abstract] OR “EWS” [Title/Abstract] OR “Early Warning and Response System*” [Title/Abstract] OR “ewart” [Title/Abstract] OR “regional data shar*” [Title/Abstract] OR “big data” [Title/Abstract] OR “bigdata” [Title/Abstract] OR “machine learning” [Title/Abstract] OR “artificial intelligence” [Title/Abstract] OR “AI” [Title/Abstract] OR “deep learning” [Title/Abstract] OR “data mining” [Title/Abstract] OR “predictive analysis” [Title/Abstract] OR “modelling” [Title/Abstract] OR “neural network” [Title/Abstract] OR “software*” [Title/Abstract] OR “simulation*” [Title/Abstract] OR “dynamic anal*” [Title/Abstract] OR “multicriter* evaluation*” [Title/Abstract]))) AND ((climat*[Title/Abstract] OR weather[Title/Abstract] OR meteorolog*[Title/Abstract] OR “atmospheric condition*”[Title/Abstract] OR “Climate change”[Title/Abstract] OR “changing climate”[Title/Abstract] OR “climate-informed”[Title/Abstract] OR “global change”[Title/Abstract] OR “cambio climático”[Title/Abstract] OR “Clima* epidemiolog*”[Title/Abstract] OR “Clima* modelling”[Title/Abstract] OR “clima* data”[Title/Abstract] OR “clima* system*”[Title/Abstract] OR “clima* service*”[Title/Abstract] OR “clima* partnership”[Title/Abstract] OR “clima* health*”[Title/Abstract] OR “clima* forecast*”[Title/Abstract] OR “clima* informa*”[Title/Abstract] OR “Extreme weather event”[Title/Abstract] OR Cyclone[Title/Abstract] OR “Extreme weather”[Title/Abstract] OR Flood*[Title/Abstract] OR “Heat wave”[Title/Abstract] OR Hurricane[Title/Abstract] OR Storm[Title/Abstract] OR Moisture[Title/Abstract] OR Altitude[Title/Abstract] OR “Dew point”[Title/Abstract] OR Humid*[Title/Abstract] OR “Saturation deficit”[Title/Abstract] OR “Soil moisture”[Title/Abstract] OR Oscillation*[Title/Abstract] OR “La Niña”[Title/Abstract] OR “El Niño”[Title/Abstract] OR “North Atlantic Oscillation”[Title/Abstract] OR “Pacific Decadal Oscillation”[Title/Abstract] OR ENSO[Title/Abstract] OR ENOS[Title/Abstract] OR “Partic* matter”[Title/Abstract] OR Dust*[Title/Abstract] OR Turbidity[Title/Abstract] OR Rain*[Title/Abstract] OR Drought[Title/Abstract] OR “Heavy rain”[Title/Abstract] OR “High rainfall”[Title/Abstract] OR Monsoon[Title/Abstract] OR Precipitation[Title/Abstract] OR “Annual rainfall”[Title/Abstract] OR Runoff[Title/Abstract] OR Wet*[Title/Abstract] OR temperature*[Title/Abstract] OR Freezing[Title/Abstract] OR “Warm years”[Title/Abstract] OR Warm*[Title/Abstract] OR Winter[Title/Abstract] OR Wind*[Title/Abstract] OR NDVI[Title/Abstract] OR Salinity[Title/Abstract] OR deforestation[Title/Abstract] OR drought[Title/Abstract] OR adaptation[Title/Abstract] OR variability[Title/Abstract]))) AND ((“Latin America”[Title/Abstract] OR “South America”[Title/Abstract] OR “Central America”[Title/Abstract] OR “Caribbean”[Title/Abstract] OR “América Latina”[Title/Abstract] OR latin*[Title/Abstract] OR “América do Sul”[Title/Abstract] OR “América Central”[Title/Abstract] OR Caribe[Title/Abstract] OR Aruba[Title/Abstract] OR Argentina[Title/Abstract] OR “Antigua y Barbuda”[Title/Abstract] OR “Antígua e Barbuda”[Title/Abstract] OR Bahamas[Title/Abstract] OR Belize[Title/Abstract] OR Bolivia[Title/Abstract] OR Brasil[Title/Abstract] OR Barbados[Title/Abstract] OR Chile[Title/Abstract] OR Colombia[Title/Abstract] OR “Costa Rica”[Title/Abstract] OR Cuba[Title/Abstract] OR “Ilhas Cayman”[Title/Abstract] OR “Cayman Islands”[Title/Abstract] OR Dominica*[Title/Abstract] OR Ecuador[Title/Abstract] OR Granada[Title/Abstract] OR Grenada[Title/Abstract] OR Guatemala[Title/Abstract] OR Guiana[Title/Abstract] OR Guyana[Title/Abstract] OR Honduras[Title/Abstract] OR Haiti[Title/Abstract] OR Jamaica[Title/Abstract] OR México[Title/Abstract] OR Nicaragua[Title/Abstract] OR Panamá[Title/Abstract] OR Peru[Title/Abstract] OR “Puerto Rico”[Title/Abstract] OR Paraguai[Title/Abstract] OR Paraguay[Title/Abstract] OR “El Salvador”[Title/Abstract] OR Suriname[Title/Abstract] OR “Saint Maarten”[Title/Abstract] OR “Ilha de Turks e Caicos”[Title/Abstract] OR “Turks and Caicos”[Title/Abstract] OR “Trinidad e Tobago”[Title/Abstract] OR Uruguay[Title/Abstract] OR “São Vicente e Granadinas”[Title/Abstract] OR “St. Vincent and the Grenadines”[Title/Abstract] OR Venezuela[Title/Abstract] OR “Ilhas Virgens”[Title/Abstract] OR “Virgin Islands”[Title/Abstract]))) NOT ((“covid*”[Title/Abstract] OR “sars*”[Title/Abstract])) [2015–2023]
References	195

## Appendix E. Search Strategies ProQuest

**Characteristic**	**Report**
Type of search	New
Database	BioRxiv, ProQuest Dissertations and Theses, ArXiv.org and MedRxiv
Platform	Proquest
Search date	20 December 2023
Date range for the search	2015–Current
**Search strategy**	noft(“climate sensitive disease*” OR “climate-sensitive disease*” OR Leishmaniasis OR Chagas OR dengue OR “yellow fever” OR malaria OR onchocerciasis OR zika OR echinococcosis OR helminthiasis OR chikungunya OR Shigellosis OR “E. coli” OR Campylobacter OR rotavirus OR cholera OR norovirus OR typhoid OR amoebiasis OR tuberculosis OR Pneumococcal OR influenza OR “Antimicrobial resistance” OR varicella OR measles OR Schistosomiasis OR Meningitis) AND noft(“information system*” OR integration OR model* OR “Health Information” OR “Data system*” OR “Decision Support System*” OR “Digital tool*” OR “Geographical Information System*” OR GIS OR “Geospatial system*” OR “Epidemiological assessment*”) AND noft(climat* OR weather OR meteorolog* OR “atmospheric condition*” OR “Climate change” OR “changing climate” OR “climate-informed” OR “global change” OR “cambio climático” OR “Extreme weather event” OR “Extreme weather” OR “Heat wave” OR ENSO OR Rain* OR Drought OR “Heavy rain” OR “High rainfall” OR Monsoon OR Precipitation OR “Annual rainfall” OR Runoff OR temperature* OR Freezing OR “Warm years” OR Warm* OR Winter OR Wind* OR NDVI OR deforestation OR drought OR adaptation) NOT noft(covid* OR sars*) AND noft(“Latin America” OR “South America” OR “Central America” OR “Caribbean” OR “Am*rica Latina” OR latin* OR “Am*rica do Sul” OR “Am*rica Central” OR Caribe OR Aruba OR Argentina OR “Antigua y Barbuda” OR Barbuda OR Bahamas OR Belize OR Bol*via OR Bra*il OR Barbados OR Chile OR Colombia OR “Costa Rica” OR Cuba OR “Ilhas Cayman” OR “Cayman Islands” OR Dominica* OR E*uador OR Granada OR Grenada OR Guatemala OR Guiana OR Guyana OR Honduras OR Haiti OR Jamaica OR M*xico OR Nicar*gua OR Panam* OR Peru OR “P*rto Rico” OR Paraguai OR Paraguay OR “El Salvador” OR Suriname OR “Saint Maarten” OR “Saint Maarten” OR “Ilha de Turks e Caicos” OR “Turks and Caicos” OR “Trinidad” OR Urugua* OR “S*o Vicente e Granadinas” OR “St. Vincent and the Grenadines” OR Venezuela OR “Ilhas Virgens” OR “Virgin Islands”) Filters: (Working Papers OR Dissertations & Theses) NOT (Scholarly Journals AND Conference Papers & Proceedings AND Reports AND Newspapers AND Wire Feeds AND Trade Journals AND Magazines AND Blogs, Podcasts, & Websites) [1 January 2015 hasta December 2023]
References	610

## Appendix F. Search Strategies PAHO IRIS

**Characteristic**	**Report**
Type of search	New
Database	PAHO IRIS
Platform	PAHO IRIS (https://iris.paho.org/)
Search date	14 December 2023
Date range for the search	2015–Current
Language restrictions	None
Other limitations	None
Search strategy	(“climate-sensitive disease” OR “climate sensitive disease” OR “climate-sensitive infect*” OR “enfermedades sensibles al clima” OR vector-borne OR “mosquito-borne” OR “Aedes aegypti” OR leishmania* OR “Chagas disease” OR trypanosom* OR dengue* OR “yellow fever” OR malaria OR zikv OR zika OR chikungunya OR waterborne OR foodborne OR shigell* OR “Escherichia coli” OR e.coli OR campylobacter OR salmonell* OR typhoid OR amoebiasis OR soilborne OR tuberculosis OR influenza OR rotavirus OR “climate-sensitive diseases” OR pneumococ* OR streptococc* OR varicella-zoster OR measles OR cholera OR norovirus OR helminth* OR meningi* OR tickborne OR Cysticerc*) AND (Economic* OR Cost* OR financ* OR Expenditure* OR “Out of pocket” OR impoverishment OR budget* OR tax* OR burden OR “healthcare spend*” OR wage* OR Salary* OR QALY* OR DALY* OR “resource allocation” OR “Willingness-to-pay” OR monetar* OR productivity OR “Policy evaluation” OR benefit* OR “Years of life lost” OR “value of statistical life” OR adaptat* OR utility OR effectiveness OR efficiency OR gasto OR presupuesto OR impuesto OR AVAC OR eficiencia OR utilidad OR efectividad) AND (climat* OR weather OR “meteorological” OR “atmospheric conditions” OR “Climate change” OR “Extreme weather events” OR Cyclone OR Flood* OR “Heat wave” OR Hurricane OR Storm OR Humidity OR “Soil moisture” OR “la Niña” OR “el Niño” OR ENSO OR “Particulate matter” OR Rain* OR Drought OR Monsoon OR Precipitation OR temperature* OR Wind* OR NDVI OR deforestation OR drought) Subject Not Contain: Communicable Diseases × Subject Not Contain: Comité Ejecutivo de la OPS × Subject Not Contain: PAHO Directing Council × Subject Not Contain: Consejo Directivo de la OPS × Subject Not Contain: Fiebre Aftosa × Subject Not Contain: COVID-19 × Subject Not Contain: Salud Pública Veterinaria × Subject Not Contain: Laboratorios × Date issued: [2015 TO 2023] × Subject Not Contain: Salud Mental × Subject Not Contain: CD54 × Subject Not Contain: Cooperación Internacional × Subject Not Contain: CD58 × Subject Not Contain: VIH × Author: Pan American Health Organization × Subject Not Contain: Cooperación Técnica × Subject Not Contain: SARS-CoV-2 × Subject Not Contain: CSP29
References	104

## Appendix G. Search Strategies IDB

**Characteristic**	**Report**
Type of search	New
Database	IDB (https://publications.iadb.org/en)
Platform	IDB (https://publications.iadb.org/en)
Search date	14 December 2023
Date range for the search	2015–Current
Language restrictions	None
Other limitations	None
Search strategy	(Climate health) (Climate sensitive diseases)
References	23

## Appendix H. Search Strategies CAF

**Characteristic**	**Report**
Type of search	New
Database	CAF
Platform	SCIOTECA (https://scioteca.caf.com/)
Search date	14 December 2023
Date range for the search	2015–Current
Language restrictions	None
Other limitations	None
Search strategy	(Climate health) (Climate sensitive diseases)
References	16

## Appendix I. Search Strategies ECLAC

**Characteristic**	**Report**
Type of search	New
Database	ECLAC
Platform	ECLAC Library (https://www.cepal.org/en/library)
Search date	14 December 2023
Date range for the search	2015–Current
Search strategy	(Climate health) AND (Climate sensitive diseases)
References	25

## Appendix J. Key Terms Used in the Text Matching and Filtering Process

**Set of Key Terms**	**Column Name**
arboviral diseases, dengue virus, dengue, Aedes, yellow fever, zika, vector-borne diseases	Arboviral diseases
*dengue, Aedes*	Dengue
*yellow fever, Aedes*	Yellow fever
*zika, Aedes*	Zika
*Leishmania, Leishmaniasis, Lutzomyia longipalpis, Nyssomyia neivai, Migonemyia migonei, phlebotominae*	Leishmaniasis
*Trypanosoma cruzi, Chagas disease, Trypanosoma, chagas*	Chagas disease
Plasmodium, malaria	malaria
*Onchocerca volvulus*, onchocerciasis	onchocerciasis
Echinocococcus, cystic echinococcosis	Cystic echinococcosis
*Ascaris lumbricoides*, *Trichuris trichiura*, hookworms, *Ancylostoma duodenale*, *Necator americanus*, helminthiasis, helminthiases, helminth infection	Helminth infection
Chikungunya	Chikungunya
shigella, Shigellosis	Shigellosis
escherichia coli, *E. coli*	*E. coli*
campylobacter, *Campylobacter enteritis*	Campylobacter enteritis
rotavirus, rotavirus infection	Rotavirus infection
vibrio cholerae, cholera	Cholera
Norovirus	Norovirus
typhoid and Paratyphoid fever	Typhoid and Paratyphoid fever
*Entamoeba histolytica*, amoebiasis	Amoebiasis
*Mycobacterium tuberculosis*, tuberculosis	Tuberculosis
*Streptococcus pneumoniae*, Pneumococcal Disease	Pneumococcal Disease
influenza viruses, influenza	Influenza
Antimicrobial resistance	Antimicrobial resistance
varicella-zoster, varicella, herpes zoster	Varicella and herpes zoster
Morbillivirus, measles	Measles
Schistosoma, Schistosomiasis	Schistosomiasis
Meningitis, Pachymeningitis, Pachymeningitides	Meningitis
salmonella, salmonelosis, salmonellosis	Salmonellosis
neural network, NN, NNAR	Neural Network
SVM, support vector machines, decision trees, gradient boosting	Supervised Learning
clustering, k-means, DBSCAN, PCA, principal component analysis, LDA, latent dirichlet allocation, embeddings	Unsupervised Learning
label propagation, self-training	Semi-supervised Learning
Q-learning, policy gradient, multiagents	Reinforcement Learning
Bayesian, Bayesian analysis, Markov-chain, MCMC	Bayesian Analysis
time series, temporal series, autoregressive, auto regressive, ARMAX, ARIMA	Time-Series Analysis
ANOVA, analysis of variance, chi-square test, t-test, statistical modeling	Statistical Tests
linear regression, Linear Model, GLM, Stepwise Linear, Multiple Linear, logistic regression, multiple logistic, poisson, Negative Binomial, beta regression	Regression Models
differential equations, ordinary differential equations, ODE, mathematical modeling, optimization, simulation, game theory	Mathematical Modeling
social network analysis, graph theory, community detection, network metrics	Social Network Analysis
Ecological niche, ENM, Species distribution, niche model, GARP, Correlative, maximum entropy, MaxEnt	Niche Modeling
Skeeter Buster, AedesBA, Cox, Kaplan–Meier	Specific Models
geostatistical, kriging, spatial interpolation, spatial distribution, Spatial Autocorrelation, Moran	Spatial Analysis
Linear Mixed Model, GLMM, Generalized Additive Model, GAM, random forests	Advanced Statistical Models
Spectral Analysis, Map Algebra	Other Models
R0, extended mathematical framework, R naught, reproduction number	Disease-Spread Models
temporal forecasting, multivariate forecast	Forecasting Models

## Appendix K. List of Articles in Each Group by Disease and Information Analysis Technique Category

**Disease**	**Analytical Method(s) and References**
Arboviral diseases	Niche Modeling [57]
Arboviral diseases; Dengue	Bayesian Analysis [58]; Disease-Spread Models [51,59]; Supervised Learning [60]
Arboviral diseases; Dengue; Chikungunya; Zika	Statistical Tests [61]
Arboviral diseases; Dengue; Chikungunya; yellow fever	Forecasting Models; Time-Series Analysis [62]
Arboviral diseases; Dengue; Zika; yellow fever; Chikungunya	Advanced Statistical Models [50]
Arboviral diseases; Dengue; yellow fever; Zika	Mathematical Modeling [63]
Arboviral diseases; Zika	Spatial Analysis [64]
Arboviral diseases; Zika; Chikungunya; yellow fever	Forecasting Models [65]
Arboviral diseases; Zika; yellow fever	Disease-Spread Models [66]
Chagas disease	Bayesian Analysis [67]; Niche Modeling [68]; Other Models [69]; Regression Models [70]; Spatial Analysis [71]; Neural Network [72]; Unsupervised Learning; Advanced Statistical Models [51]
Chikungunya	Disease-Spread Models [73]; Disease-Spread Models; Mathematical Modeling [74]; Neural Network [75]; Regression Models [52]; Unsupervised Learning [76]
Chikungunya; Zika	Regression Models; Supervised Learning [53]
Cholera	Niche Modeling [77]
Dengue	Spatial Analysis [78]
Dengue; Arboviral diseases	Time-Series Analysis; Neural Network [79]; Unsupervised Learning [80]
Dengue; Arboviral diseases; yellow fever; Zika	Regression Models [81]
Dengue; Zika; yellow fever; Chikungunya	Niche Modeling [82]
Influenza	Regression Models [83]
Leishmaniasis	Advanced Statistical Models [84]; Niche Modeling; Supervised Learning [85]; Regression Models [86]; Spatial Analysis [87]; Specific Models [88]; Supervised Learning; Time-Series Analysis [54]
Leishmaniasis; Chagas disease	Bayesian Analysis; Mathematical Modeling [89]
Rotavirus infection	Time-Series Analysis [67]
Salmonellosis	Time-Series Analysis; Spatial Analysis [90]
Schistosomiasis	Niche Modeling; Spatial Analysis [90,91]; Unsupervised Learning [10]
Zika	Neural Network [92]
Zika; Chikungunya	Bayesian Analysis; Unsupervised Learning [92]
Malaria	Advanced Statistical Models [93]; Bayesian Analysis; Mathematical Modeling; Spatial Analysis [55]; Niche Modeling [94]; Statistical Tests; Regression Models [95]; Time-Series Analysis; Unsupervised Learning [96]
Yellow fever	Forecasting Models [97,98]
Yellow fever; Chikungunya	Neural Network [99]; Spatial Analysis [100]
Yellow fever; Zika	Bayesian Analysis [56]; Time-Series Analysis [101]
